# Magnetothermally-controllable coating for scattering and absorption in the terahertz spectral regime

**DOI:** 10.1016/j.heliyon.2024.e26835

**Published:** 2024-02-28

**Authors:** Hamad M. Alkhoori, Noura N. Alderei, Aryam S. Alshamsi

**Affiliations:** United Arab Emirates University, Department of Electrical Engineering, Al Ain, United Arab Emirates

## Abstract

Plane-wave scattering and absorption characteristics of a spherical core composed of an isotropic material, and covered by InSb spherical coating are investigated in the terahertz spectral regime. The InSb coating is subjected to a magnetostatic field; hence it is a gyrotropic, uniaxial dielectric material comprised of two parameters: temperature and magnetostatic field. It is seen that the interplay of these two parameters can modify the various scattering efficiencies, depending on (i) the frequency of the incident plane wave, (ii) the incident plane-wave orientation with respect to the magnetostatic field's direction, and (iii) the identity of the core material. In particular, for a fixed orientation of the incident plane wave, the impact of the temperature and the magnetostatic field on the total scattering and the backscattering efficiencies becomes more pronounced at higher frequencies, compared to lower frequencies. The absorption efficiency, on the other hand, is strongly dependent upon these two parameters, regardless of the frequency. On fixing the two parameters of InSb, the role of the orientation of the incident plane wave with respect of the magnetostatic field' direction emerges in some spectral regimes for the total scattering and absorption efficiencies, and in high-frequency spectral regimes for the backscattering efficiency. These hold true for dielectric core, as well as for conductive core.

## 1 Introduction

Scattering of plane waves by objects composed of exotic materials is a recurring topic in electromagnetics literature [1], [2], [3]. This can be attributed to the availability of homogenization theorems, in which a compound material can be conceptualized as a mixture of two or more electrically small constituents [4], [5], [6], [7], [8], [9], [10]. Moreover, some electromagnetically simple materials transform into exotic ones when subjected to a biasing factor. Examples are plasmas, ferrites, and indium antimonide (InSb) when subjected to a magnetostatic field [11], [12]. In particular, in the absence of the magnetostatic field, these materials are isotropic. They become anisotropic (i.e., exhibiting directionality properties) when exposed to a magnetostatic field.

Isotropic and anisotropic materials can be used as a coating layer for a core object [13], [14]. As compared to isotropic materials, anisotropic materials provide more input parameters [5], each in which could be used to control scattering and absorption characteristics of the core in a desired way. For instance, absorption of the core can be reduced for protection from heating due to exposure to an incident electromagnetic wave [15]. This has been accomplished using an isotropic InSb coating, which has proven to be effective in absorption and extinction reduction for dielectric cores by varying the temperature parameter in the terahertz spectral regime [15]. Also, belonging to radar applications, the backscattering of the core can be reduced; thereby making it invisible, or barely visible, for monostatic detection [16], [17], [18], [19]. Backscattering reduction by plasmas and ferrites has been employed before, primarily for metallic cores. A plasma-based artificial magnetic conductor has been used to reduce backscattering efficiency for planar, cylindrical, and spherical metallic surfaces [20]. Also, a microstrip patch antenna, when loaded by magnetized ferrite, has displayed some reduction in backscattering efficiency as compared to when loaded with conventional dielectric materials [21]. Moreover, isotropic InSb coating has proven to be effective in absorption and extinction reduction for dielectric cores by varying the temperature parameter [15]. Whereas plasmas and ferrites have been utilized in the gigahertz spectral regime, InSb becomes functional only in the terahertz spectral regime, as discussed elsewhere [15].

Terahertz spectral regime lies between microwave and infrared spectral regimes (i.e., frequencies ranging between 0.1 to 20 THz [22]). Electromagnetic waves radiating in the terahertz spectral regime can exist naturally (e.g., black-body radiation [23]), or they can exist artificially from man-made devices (e.g., backward wave oscillator (BWO) [24], infrared laser (FIR laser), and quantum cascade laser [25]). Terahertz radiation can be made beneficial for satellite communication systems [26], [27] due to large bandwidth requirements. Also, it can be employed in medical industry, for instance, in cancer treatments [28], [29], [30], [31]. Due to the interrelation between InSb material and terahertz radiation, InSb has proved to have potential applications in the terahertz spectral regime. For example, InSb was employed in designing perfect absorbers used for sensing applications [32], [33], [34]. Moreover, InSb was employed in many other disciplines, including thermal emission, imaging, radar applications, and optoelectronic devices [35], [36], [37], [38], [39], [40].

In the terahertz spectral regime, InSb is isotropic dielectric with thermally controllable properties. When, additionally, exposed to a magnetostatic field, InSb becomes uniaxial dielectric endowed with gyrotropy [11]. In this perspective, InSb becomes magnetothermally controllable (i.e., acquired with two parameters: temperature, and magnetostatic field) in the terahertz spectral regime [12], [41]. This could make InSb suitable in applications involving anisotropic materials, such as polarization conversion, asymmetric transmission [42], beam splitting [43], and lensing [44].

The temperature character of the isotropic-version InSb was investigated in absorption and extinction characteristics [15]. In this work, we propose to investigate the temperature and magnetostatic field characters of the anisotropic-version InSb in scattering and absorption characteristics. In particular, we consider a spherical core (dissipated dielectric or conductive) covered by a conformal spherical InSb coating, and illuminated by a plane wave in the terahertz spectral regime. On varying (i) the incident plane wave orientation with respect to the magnetostatic field direction, (ii) the temperature of the InSb coating, and (iii) the magnitude of the magnetostatic field of the InSb coating, we capture the effects of the aforementioned parameters on the total scattering, absorption, and backscattering efficiencies of the core-coat structure.

The plan of this paper is as follows. We describe the boundary-value problem in Sec. 2. Numerical results on the frequency-dependent $Q_{s}$, $Q_{a}$, and $Q_{b}$ in relation to the temperature and magnetostatic field are presented in Sec. 3. Conclusions are presented in Sec. 4.

An exp⁡(−i2πft) dependence on time *t* is implicit throughout this work with $i=\sqrt{-1}$ and *f* as the linear frequency. The permittivity and the permeability of free space (i.e., vacuum) are denoted by $\varepsilon_{0}$ and $\mu_{0}$, respectively, whereas $k_{0}=2\pi f\sqrt{\mu_{0}\varepsilon_{0}}$ and $\eta_{0}=\sqrt{\mu_{0}/\varepsilon_{0}}$ are the wavenumber in and the intrinsic impedance of free space, respectively. Vectors are in boldface, $\left\{\overset{ˆ}{x},\overset{ˆ}{y},\overset{ˆ}{z}\right\}$ is the triad of unit Cartesian vectors, and dyadics are double underlined.

## 2 Theory

Consider a sphere of radius *a* centered about the origin, and coated by a concentric sphere of radius *b*. The region r<a is refereed to as the core sphere, and is occupied by a linear, homogeneous medium of relative permittivity $\varepsilon_{\text{core}}$. The region a<r<b is referred to as the coating layer, and is occupied by InSb of complex relative permittivity dyadic ${\underline{\underline{\varepsilon }}}_{\text{InSb}}$. Finally, the region r>b is vacuous.

Without any loss of generality, we let the magnetostatic field be directed along the *x* axis (i.e., $B_{o}=B_{o}\overset{ˆ}{x}$). Then, the relative permittivity dyadic ${\underline{\underline{\varepsilon }}}_{\text{InSb}}$ becomes [11]

(1) $${\underline{\underline{\varepsilon }}}_{\text{InSb}}=\varepsilon_{\mathrm{xx}}\overset{ˆ}{x}\overset{ˆ}{x}+\varepsilon_{\mathrm{yy}}(\overset{ˆ}{y}\overset{ˆ}{y}+\overset{ˆ}{z}\overset{ˆ}{z})+\varepsilon_{\mathrm{yz}}(\overset{ˆ}{y}\overset{ˆ}{z}-\overset{ˆ}{z}\overset{ˆ}{y}),$$

where $\overset{ˆ}{x}\overset{ˆ}{x}$, etc., are dyads. The scalars $\varepsilon_{\mathrm{xx}}$, $\varepsilon_{\mathrm{yy}}$, and $\varepsilon_{\mathrm{yz}}$ appearing in Eq. (1) are given by [12], [41]

(2) $$\varepsilon_{\mathrm{xx}}=\varepsilon_{\infty }-q_{e}^{2}\left(\frac{n_{i}}{2\pi f\varepsilon_{0}m}\right)\frac{1}{2\pi f+i\gamma },$$

(3) $$\varepsilon_{\mathrm{yy}}=\varepsilon_{\infty }-q_{e}^{2}\left(\frac{n_{i}}{2\pi f\varepsilon_{0}m}\right)\frac{2\pi f+i\gamma }{{\left(2\pi f+i\gamma \right)}^{2}-{\left(\frac{q_{e}B_{o}}{m}\right)}^{2}},$$

and

(4) $$\varepsilon_{\mathrm{yz}}=iq_{e}^{2}\left(\frac{n_{i}}{2\pi f\varepsilon_{0}m}\right)\frac{\left(\frac{q_{e}B_{o}}{m}\right)}{{\left(2\pi f+i\gamma \right)}^{2}-{\left(\frac{q_{e}B_{o}}{m}\right)}^{2}}.$$

In Eqs. (2)-(4), $\varepsilon_{\infty }=15.68$ is the high-frequency limit, $m=0.015m_{e}$ with $m_{e}=9.109\times 10^{-31}$ kg as the rest mass of an electron, $q_{e}=-1.602\times 10^{-19}$ C is the charge of an electron, $\gamma =\pi \times 10^{11}$ rad s^−1^ is the damping constant, and

(5) $$n_{i}=5.76\times 10^{20}T^{3/2}\exp\left(-\frac{E_{g}}{2k_{B}T}\right)$$

is the temperature-dependent intrinsic carrier density (in m^−3^) [45]. In Eq. (5), $E_{g}=0.26$ eV is the bandgap energy of InSb, and $k_{B}=8.617\times 10^{-5}$ eV K^−1^ is the Boltzmann constant.

### 2.1 Incident field

Let the coated sphere be illuminated by an incident plane wave, whose electromagnetic field phasors are given by

(6) $$\begin{array}{c} E_{\text{i}}(r,f)={\overset{ˆ}{e}}_{\text{i}}\exp(ik_{0}{\overset{ˆ}{k}}_{\text{i}}\cdot r)\\ H_{\text{i}}(r,f)=\eta_{0}^{-1}\;{\overset{ˆ}{h}}_{\text{i}}\exp(ik_{0}{\overset{ˆ}{k}}_{\text{i}}\cdot r) \end{array}\}\;.$$

In Eqs. (6), the unit vector ${\overset{ˆ}{e}}_{\text{i}}$ denotes the polarization state, the unit vector ${\overset{ˆ}{k}}_{\text{i}}$ denotes the propagation direction, with the constraint ${\overset{ˆ}{k}}_{\text{i}}\cdot {\overset{ˆ}{e}}_{\text{i}}=0$, and ${\overset{ˆ}{h}}_{\text{i}}={\overset{ˆ}{k}}_{\text{i}}\times {\overset{ˆ}{e}}_{\text{i}}$.

### 2.2 Scattered field

In the region r>b (i.e., outside the coated sphere), the total electromagnetic field phasors are given by

(7) $$\begin{array}{c} E(r,f)=E_{\text{i}}(r,f)+E_{\text{s}}(r,f)\\ H(r,f)=H_{\text{i}}(r,f)+H_{\text{s}}(r,f) \end{array}\}\;,\;r>b\;,$$

where $E_{\text{s}}(r,f)$ and $H_{\text{s}}(r,f)$ are the scattered electromagnetic field phasors. These scattered electromagnetic field phasors were determined using the finite-element method implemented in HFSS software [46].

### 2.3 Scattering and absorption efficiencies

The scattered electromagnetic field phasors introduced in Eqs. (7) can be used to calculate the scattered Poynting vector $S_{\text{s}}(r,f)$, given by

(8) $$S_{\text{s}}(r,f)=\frac{1}{2}\text{Re}\{E_{\text{s}}(r,f)\times H_{\text{s}}^{⁎}(r,f)\},$$

where $\text{Re}\{\;{}^{•}\;\}$ is the real part and the asterisk (⁎) denotes the complex conjugate. Then, the total scattered power $P_{\text{s}}(f)$ can be calculated as [47]

(9) $$P_{\text{s}}(f)=\int_{S_{\infty }}S_{\text{s}}(r,f)\;{}^{•}\;\overset{ˆ}{n}(r)\;d^{2}r,$$

where $S_{\infty }$ is a surface of an infinite extent, $\overset{ˆ}{n}(r)$ is the unit inward normal on $S_{\infty }$, and $d^{2}r$ is the differential surface element of $S_{\infty }$.

Using the scattered Poynting vector defined in Eq. (8), the backscattered power $P_{\text{b}}(f)$ can be obtained as

(10) $$P_{\text{b}}(f)=\lim_{r\to \infty }S_{\text{s}}(-{\overset{ˆ}{k}}_{\text{i}},f)\;{}^{•}\;r^{2}\overset{ˆ}{n}(-{\overset{ˆ}{k}}_{\text{i}}).$$

Due to the losses associated with the mediums occupying the core sphere and the coating layer, there will be an amount of power from the incident plane wave that is being absorbed. The absorbed Poynting vector $S_{\text{a}}(r,f)$, quantifying that amount, is given by

(11) $$S_{\text{a}}(r,f)=-\frac{1}{2}\text{Re}\{E(r,f)\times H^{⁎}(r,f)\}.$$

Then, the total absorbed power can be obtained using Eq. (11) as [47]

(12) $$P_{\text{a}}(f)=\int_{S_{\text{coat}}}S_{\text{a}}(r,f)\;{}^{•}\;\overset{ˆ}{n}(r)\;d^{2}r,$$

where $S_{\text{coat}}$ is the surface of the coated sphere, and $\overset{ˆ}{n}(r)$ is the unit outward normal on $S_{\text{coat}}$.

In scattering literature, the scattering and absorption characteristics of a scatterer are quantified by virtue of efficiencies, rather than power. The total scattering efficiency $Q_{s}$ can be related to the total scattered power in Eq. (9) through [48]

(13) $$Q_{s}(f)=\frac{2\eta_{0}}{\pi b^{2}}P_{\text{s}}(f).$$

Similarly, the backscattered efficiency $Q_{b}$ can be obtained from Eq. (10) as

(14) $$Q_{b}(f)=\frac{2\eta_{0}}{\pi b^{2}}P_{\text{b}}(f),$$

and the absorption efficiency $Q_{a}$ from Eq. (12) as

(15) $$Q_{a}(f)=\frac{2\eta_{0}}{\pi b^{2}}P_{\text{a}}(f).$$

## 3 Numerical results and discussion

We now numerically study the effects of the InSb coating in $Q_{s}$, $Q_{a}$, and $Q_{b}$. These efficiencies depend, in general, on

• the frequency of the incident plane wave (*f*),
• the incident plane wave orientation (${\overset{ˆ}{k}}_{\text{i}}$, ${\overset{ˆ}{e}}_{\text{i}}$, and ${\overset{ˆ}{h}}_{\text{i}}$) relative to the magnetostatic field orientation (${\overset{ˆ}{B}}_{o}$),
• the core radius (*a*) and the coating's thickness (b−a),
• the relative permittivity of the core medium [$\varepsilon_{\text{core}}(f)$], as well as
• the magnetostatic field ($B_{o}$) and the temperature (*T*) of the coating.

For all numerical results presented in this section, we fixed a=47.7 μm and b=1.2a.

For the sake of illustration, we investigated the dependences of $Q_{s}$, $Q_{a}$, and $Q_{b}$ on $B_{o}$ and *T* in the spectral regime f∈[0.1,4] THz, when (i) the magnetostatic field is parallel to the propagation direction of the incident plane wave (i.e., ${\overset{ˆ}{B}}_{o}∥\pm {\overset{ˆ}{k}}_{\text{i}}$), (ii) the magnetostatic field is parallel to the electric field direction of the incident plane wave (i.e., ${\overset{ˆ}{B}}_{o}∥\pm {\overset{ˆ}{e}}_{\text{i}}$), and (iii) the magnetostatic field is parallel to the magnetic field direction of the incident plane wave (i.e., ${\overset{ˆ}{B}}_{o}∥\pm {\overset{ˆ}{h}}_{\text{i}}$). This was done when the core material is (i) dissipative dielectric, and (ii) perfect electric conductor (PEC).

### 3.1 Dissipative dielectric core

We let the core be made of a dissipative dielectric material described by $\varepsilon_{\text{core}}=7.6+i0.5$. The real part of the permittivity of this artificial material is close to that of silicon nitride Si_3_N_4_ ($\varepsilon_{r}=7.6+i0.06$) [49], whereas its imaginary part is substantially larger. This choice was made intentionally so as to examine the capability of InSb coating for absorption reduction for highly-absorbing cores.

#### 3.1.1 Fixed orientation

**Figure 1:**
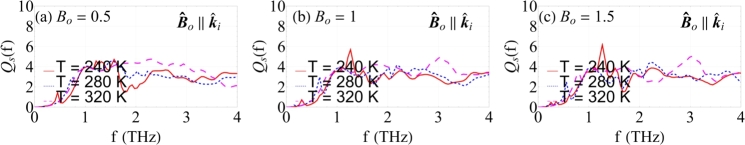
*Q*_*s*_ versus *f* ∈ [0.1,4] THz when ${\overset{ˆ}{k}}_{\text{i}}=\overset{ˆ}{x}$ and ${\overset{ˆ}{e}}_{\text{i}}=\overset{ˆ}{z}$, and the core is made of a dissipative dielectric material (*ε*_core_ = 7.6 + *i*0.5). The red solid line represents *T* = 240 K, the blue dotted line represents *T* = 280 K, and the magenta dashed line represents *T* = 320 K. (a) *B*_*o*_ = 0.5 T, (b) *B*_*o*_ = 1 T, and (c) *B*_*o*_ = 1.5 T.

In order to quantify the effects of $B_{o}$ and *T* for a fixed orientation of the incident plane wave, we set ${\overset{ˆ}{k}}_{\text{i}}=\overset{ˆ}{x}$ and ${\overset{ˆ}{e}}_{\text{i}}=\overset{ˆ}{z}$, such that ${\overset{ˆ}{B}}_{o}∥\pm {\overset{ˆ}{k}}_{\text{i}}$. Figs. 1(a)-1(c) show plots of $Q_{s}$ versus f∈[0.1,4] THz when T∈{240,280,320} K and $B_{o}\in \{0.5,1,1.5\}$ T. When $B_{o}$ is low [see Fig. 1(a)], the dependence of the total scattering efficiency on the temperature becomes virtually visible when f>0.4 THz. As $B_{o}$ increases [see Figs. 1(b) and (c)], this dependence shows up at a smaller frequency f>0.2 THz. Also, at a fixed $B_{o}$, the total scattering efficiency becomes more sensitive to the temperature at some frequencies compared to others. This behavior changes in an unpredictable manner when $B_{o}$ is changed. When T=240 K [see the red curve in Fig. 1], the dependence of the total scattering efficiency on $B_{o}$ becomes virtually visible when f>0.2 THz, whereas when T=320 K [see the magenta curve in Fig. 1], this dependence shows up when f>0.8 THz. Also, at a fixed *T*, the total scattering efficiency becomes more sensitive to $B_{o}$ at some frequencies compared to others. Like $B_{o}$, this behavior changes in an unpredictable manner when *T* is changed.

We repeated calculations when ${\overset{ˆ}{B}}_{o}∥-{\overset{ˆ}{k}}_{\text{i}}$ (not shown), and we found that, for all f∈[0.1,4] THz, T∈{240,280,320} K, and $B_{o}\in \{0.5,1,1.5\}$ T, the total scattering efficiency $Q_{s}$ when ${\overset{ˆ}{B}}_{o}∥{\overset{ˆ}{k}}_{\text{i}}$ is indistinguishable from that when ${\overset{ˆ}{B}}_{o}∥-{\overset{ˆ}{k}}_{\text{i}}$. This also holds for the cases ${\overset{ˆ}{B}}_{o}∥{\overset{ˆ}{e}}_{\text{i}}$ and ${\overset{ˆ}{B}}_{o}∥-{\overset{ˆ}{e}}_{\text{i}}$, and ${\overset{ˆ}{B}}_{o}∥{\overset{ˆ}{h}}_{\text{i}}$ and ${\overset{ˆ}{B}}_{o}∥-{\overset{ˆ}{h}}_{\text{i}}$ (not shown).

**Figure 2:**
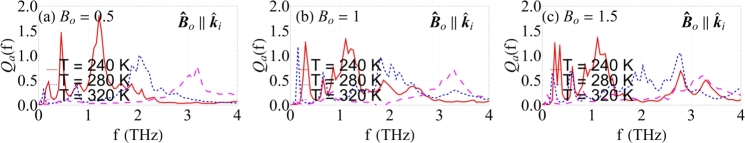
Same as Fig. 1, but for *Q*_*a*_.

Next, we examine the absorption efficiency. Figs. 2(a)-2(c) show plots of $Q_{a}$ versus f∈[0.1,4] THz when T∈{240,280,320} K, $B_{o}\in \{0.5,1,1.5\}$ T, and ${\overset{ˆ}{k}}_{\text{i}}=\overset{ˆ}{x}$ and ${\overset{ˆ}{e}}_{\text{i}}=\overset{ˆ}{z}$ (i.e., ${\overset{ˆ}{B}}_{o}∥{\overset{ˆ}{k}}_{\text{i}}$). When T=240 K, the overall behavior of $Q_{a}$ is in general oscillatory for all $B_{o}$, especially when f<1.5 THz. Also, increasing $B_{o}$ increases this oscillatory behavior in that frequency range. As *T* increases, this oscillatory behavior diminishes for all $B_{o}$. Also, note that the dependence of $Q_{a}$ on $B_{o}$ is so weak when T=320 K, compared to when T=240 K. By adjusting $B_{o}$ and *T*, $Q_{a}$ can be reduced to low levels in certain frequency ranges. It can be seen from the results that, the general trend is, $Q_{a}$ deceases as *T* increases.

Like for $Q_{s}$, we repeated calculations when ${\overset{ˆ}{B}}_{o}∥-{\overset{ˆ}{k}}_{\text{i}}$ (not shown), and we found that the absorption efficiency $Q_{a}$ when ${\overset{ˆ}{B}}_{o}∥{\overset{ˆ}{k}}_{\text{i}}$ is indistinguishable from that when ${\overset{ˆ}{B}}_{o}∥-{\overset{ˆ}{k}}_{\text{i}}$. This also holds for the cases ${\overset{ˆ}{B}}_{o}∥{\overset{ˆ}{e}}_{\text{i}}$ and ${\overset{ˆ}{B}}_{o}∥-{\overset{ˆ}{e}}_{\text{i}}$, and ${\overset{ˆ}{B}}_{o}∥{\overset{ˆ}{h}}_{\text{i}}$ and ${\overset{ˆ}{B}}_{o}∥-{\overset{ˆ}{h}}_{\text{i}}$ (not shown).

**Figure 3:**
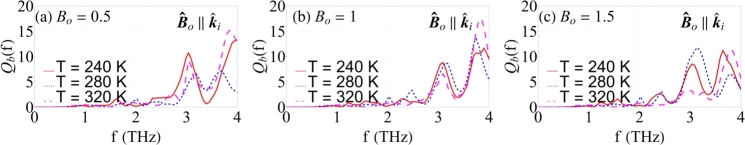
Same as Fig. 1, but for *Q*_*b*_.

Now, we examine the backscattering efficiency. Figs. 3(a)-3(c) show plots of $Q_{b}$ versus f∈[0.1,4] THz when T∈{240,280,320} K, $B_{o}\in \{0.5,1,1.5\}$ T, and ${\overset{ˆ}{k}}_{\text{i}}=\overset{ˆ}{x}$ and ${\overset{ˆ}{e}}_{\text{i}}=\overset{ˆ}{z}$ (i.e., ${\overset{ˆ}{B}}_{o}∥{\overset{ˆ}{k}}_{\text{i}}$). The backscattering efficiency is generally low at low frequencies, and then it starts oscillating as the frequency increases, regardless of *T* and $B_{o}$. Like $Q_{a}$, by adjusting $B_{o}$ and *T*, $Q_{b}$ can be reduced to low levels in certain frequency range. Notice that $Q_{b}$ is more sensitive to $B_{o}$ when T=280 K, compared to when T=240 K and T=320 K. On the other hand, $Q_{b}$ is sensitive to *T*, regardless of $B_{o}$.

**Figure 4:**
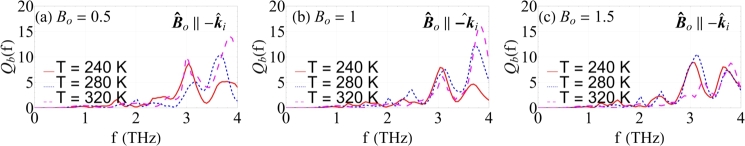
Same as Fig. 3, but when ${\overset{ˆ}{k}}_{\text{i}}∥-{\overset{ˆ}{B}}_{o}$.

We repeated the same calculations when ${\overset{ˆ}{B}}_{o}∥-{\overset{ˆ}{k}}_{\text{i}}$ in Figs. 4(a)-4(c). Co-parallel and anti-parallel configurations are now distinguishable, specially at high *f*. This is evident at low *T*, regardless of $B_{o}$. $Q_{b}$ can be further reduced when ${\overset{ˆ}{B}}_{o}∥-{\overset{ˆ}{k}}_{\text{i}}$ compared to when ${\overset{ˆ}{B}}_{o}∥{\overset{ˆ}{k}}_{\text{i}}$, or vice versa, depending on *T* and $B_{o}$. This also holds for the cases ${\overset{ˆ}{B}}_{o}∥{\overset{ˆ}{e}}_{\text{i}}$ and ${\overset{ˆ}{B}}_{o}∥-{\overset{ˆ}{e}}_{\text{i}}$, and ${\overset{ˆ}{B}}_{o}∥{\overset{ˆ}{h}}_{\text{i}}$ and ${\overset{ˆ}{B}}_{o}∥-{\overset{ˆ}{h}}_{\text{i}}$ (not shown), except that the deviation between co-parallel and anti-parallel cases is more pronounced in the ${\overset{ˆ}{B}}_{o}∥\pm {\overset{ˆ}{h}}_{\text{i}}$ case.

#### 3.1.2 Varied orientation

**Figure 5:**
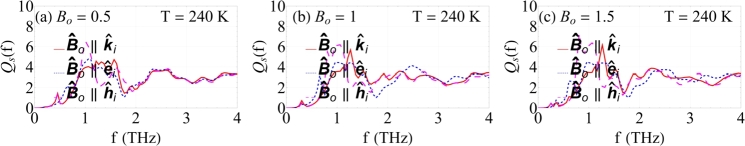
*Q*_*s*_ versus *f* ∈ [0.1,4] THz when *T* = 240 K and the core is made of a dissipative dielectric material (*ε*_core_ = 7.6 + *i*0.5). The red solid line represents $B_{o}∥{\overset{ˆ}{k}}_{\text{i}}$ incidence case, the blue dotted line represents $B_{o}∥{\overset{ˆ}{e}}_{\text{i}}$ incidence case, and the magenta dashed line represents $B_{o}∥{\overset{ˆ}{h}}_{\text{i}}$ incidence case. (a) *B*_*o*_ = 0.5 T, (b) *B*_*o*_ = 1 T, and (c) *B*_*o*_ = 1.5 T.

Now, in order to examine the effect of orientation of the incident plane wave on the efficiencies, we present the efficiencies for the cases ${\overset{ˆ}{B}}_{o}∥\pm {\overset{ˆ}{k}}_{\text{i}}$, ${\overset{ˆ}{B}}_{o}∥\pm {\overset{ˆ}{e}}_{\text{i}}$, and ${\overset{ˆ}{B}}_{o}∥\pm {\overset{ˆ}{h}}_{\text{i}}$. For the total scattering and absorption efficiencies, we only present results for co-parallel incidence cases, since co-parallel and anti-parallel incidence cases are indistinguishable. Figs. 5(a)-5(c) show plots of $Q_{s}$ versus f∈[0.1,4] THz when T=240 K and $B_{o}\in \{0.5,1,1.5\}$ T, for the cases $B_{o}∥{\overset{ˆ}{k}}_{\text{i}}$, $B_{o}∥{\overset{ˆ}{e}}_{\text{i}}$, and $B_{o}∥{\overset{ˆ}{h}}_{\text{i}}$. The total scattering efficiency is a weak function of the incidence orientation when f<0.2 THz. Then, it becomes strongly dependent upon the incidence orientation in the frequency range f∈[0.2,2] THz, regardless of $B_{o}$. Beyond that, its dependence on incidence orientation becomes dependent upon $B_{o}$. In particular, when $B_{o}=0.5$ T, $Q_{s}$ is almost independent of the incidence orientation, whereas its dependence shows up as $B_{o}$ increases.

**Figure 6:**
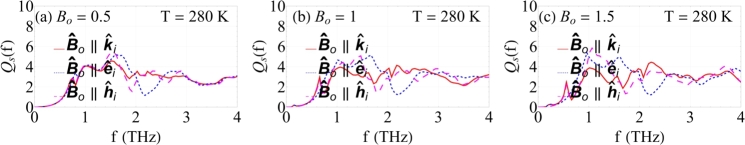
Same as Fig. 5, but when *T* = 280 K.

**Figure 7:**
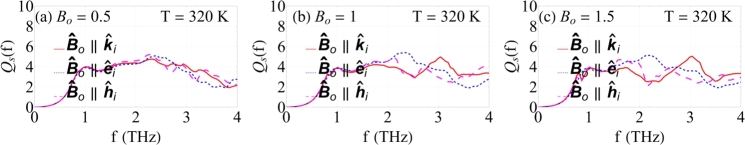
Same as Fig. 5, but when *T* = 320 K.

In order to examine the behavior at different temperatures, we present results when T=280 K and T=320 K in Figs. 6(a)-6(c) and 7(a)-7(c), respectively. As the temperature increases, the frequency range where the dependence of $Q_{s}$ on the incidence orientation becomes more pronounced, shifts. This is visibly apparent for all $B_{o}\in \{0.5,1,1.5\}$ T.

We repeated the aforementioned calculations for the absorption efficiency (not shown), and we found that the same behavior is shared by $Q_{a}$. That is, at certain frequency range, $Q_{a}$ becomes strongly dependent upon the incidence orientation, and that this frequency range shifts with the increase of temperature.

**Figure 8:**
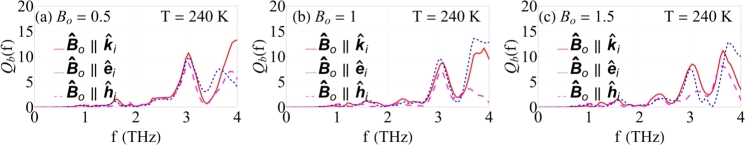
Same as Fig. 5, but for *Q*_*b*_.

**Figure 9:**
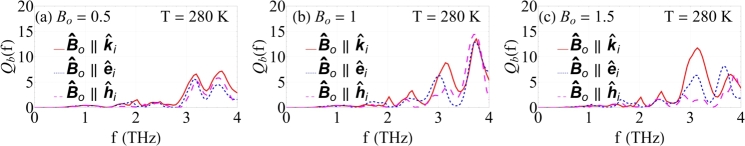
Same as Fig. 8, but when *T* = 280 K.

**Figure 10:**
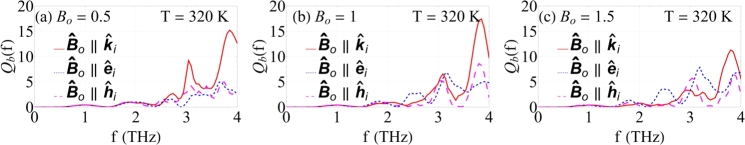
Same as Fig. 8, but when *T* = 320 K.

The backscattering efficiency, on the other hand, behaves differently. Figs. 8(a)-8(c), 9(a)-9(c), and 10(a)-10(c) show plots of $Q_{b}$ when T=240 K, T=280 K, and T=320 K, respectively. We can see that, when f<2 THz, $Q_{b}$ is a very weak function of the incidence orientation, regardless of $B_{o}$ and *T*. Beyond that, $B_{o}$ and *T* matter. In particular, when T=240 K, $Q_{b}$ becomes more sensitive to the incidence orientation as $B_{o}$ increases. This holds true for the cases T=280 and T=320. We notice that $Q_{b}$ is generally high at high frequencies and temperature when $B_{o}∥{\overset{ˆ}{k}}_{\text{i}}$, compared to when $B_{o}⊥{\overset{ˆ}{k}}_{\text{i}}$. Finally, we notice that, when either $B_{o}$ or *T* is high, at a certain frequency and for each incidence orientation, there is a combination of $B_{o}$ and *T* which makes $Q_{b}$ small.

For the anti-parallel incidence orientation case (not shown), the same conclusions of co-parallel incidence orientation case hold, except that $Q_{b}$ can be reduced further compared to co-parallel incidence orientation case.

Finally, we notice that even when $B_{o}$ is small, the backscattering efficiency is more sensitive to the incidence orientation compared to the total scattering and absorption efficiencies.

### 3.2 PEC core

**Figure 11:**
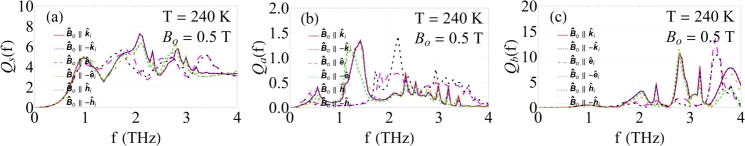
(a) *Q*_*s*_, (b) *Q*_*a*_, and (c) *Q*_*b*_ vs. *f* ∈ [0.1,4] THz when *T* = 240 K and *B*_*o*_ = 0.5 T.

**Figure 12:**
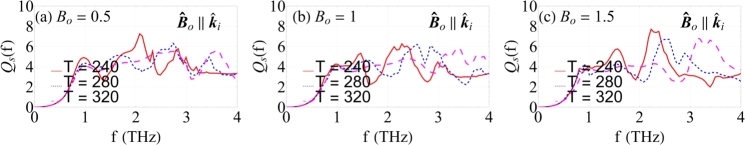
Same as Fig. 1, but for the PEC-core case.

**Figure 13:**
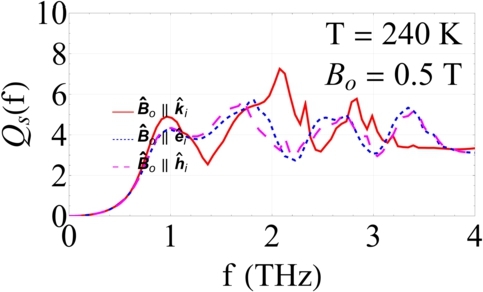
Same as Fig. 5(a), but for the PEC-core case.

Next, we let the core be made of perfect electric conductor (PEC). Although PECs may not exist in reality, good metals can be approximated by PECs [48]. In the dielectric-core case, we have seen that co-parallel and anti-parallel orientations are distinguishable only in the backscattering efficiency case. In the PEC case, on the other hand, co-parallel and anti-parallel orientations can become distinguishable in all scattering efficiencies when $B_{o}⊥{\overset{ˆ}{k}}_{\text{i}}$. This is depicted in Figs. 11(a)-11(c) for all incidence orientations when T=240 K and $B_{o}=0.5$ T. This holds true for all other combinations of *T* and $B_{o}$, except that co-parallel and anti-parallel orientations become distinguishable in the backscattering efficiency for the $B_{o}∥{\overset{ˆ}{k}}_{\text{i}}$ case when $B_{o}\in \{1,1.5\}$ T. By comparing Figs. 11(a) with 1(a), we can see that the total scattering efficiency $Q_{s}$ in the PEC-core case is almost independent on *T* at low frequencies (i.e., f<0.5 THz), compared to the dielectric-core case. This holds true for other values of $B_{o}$ (not shown). The behavior of $Q_{s}$ for the PEC-core case differs from that of the dielectric-core case in three aspects. The total scattering efficiency $Q_{s}$ is more sensitive to $B_{o}$ in the PEC-core case than in the dielectric-core case. Also, it is almost independent of *T* at low frequencies (i.e., f<0.5 THz) in the PEC-core case than in the dielectric-core case. These are depicted in Figs. 12(a)-12(c), which can be compared with Fig. 1, and it holds true for other incidence orientations (not shown). Finally, the orientation effect when $B_{o}$ is small can be captured in the PEC-core case at high frequencies as opposed to the dielectric-core case. This can be seen by comparing Figs. 13 with 5(a). This holds true, regardless of *T* (not shown). The behavior of the absorption efficiency $Q_{a}$ and backscattering efficiency $Q_{b}$ in the PEC-core case is very similar to that of the dielectric-core case, except that further reduction in $Q_{a}$ or $Q_{b}$ can be achieved by properly choosing between co-parallel and anti-parallel configurations, by virtue of Fig. 11.

## 4 Concluding remarks

We studied plane-wave scattering and absorption characteristics for a spherical scatterer covered by InSb coating using the finite-element method available in the commercially available HFSS software. Numerical results were obtained for a dielectric core and for a conductive core, focusing on the effects of (i) the temperature of the coating, (ii) the magnetostatic field of the coating, and (iii) the orientation of the incident plane wave with respect to the magnetostatic field of the coating. These were examined in the spectral regime f∈[0.1,4] THz.

As results revealed, judicious choices of *T* and $B_{o}$ can raise/lower $Q_{s}$ and $Q_{b}$ at higher frequencies, whereas raising/ lowering $Q_{a}$ can be attained at all frequencies, regardless of the orientation of the incident plane wave. This indicates that absorption reduction can be achieved at wider frequency ranges, as compared to backscattering reduction, which can be achieved at higher frequency range only. This holds for both dielectric-core case, and PEC-core case.

As for the co-parallel orientation and anti-parallel orientation states of the incident plane wave, for dielectric-core case, co-parallel ($B_{o}∥{\overset{ˆ}{k}}_{\text{i}}$) and anti-parallel ($B_{o}∥-{\overset{ˆ}{k}}_{\text{i}}$) orientation states are distinguishable only in $Q_{b}$, but not in $Q_{s}$ and $Q_{a}$. This holds true for $B_{o}∥{\overset{ˆ}{e}}_{\text{i}}$ and $B_{o}∥-{\overset{ˆ}{e}}_{\text{i}}$ orientation states, as well as for $B_{o}∥{\overset{ˆ}{h}}_{\text{i}}$ and $B_{o}∥-{\overset{ˆ}{h}}_{\text{i}}$ orientation states. Hence, backscattering reduction can be achieved not only by choosing appropriate *T* and $B_{o}$, but also by choosing between co-parallel orientation and anti-parallel orientation states. For PEC-core, on the other hand, $B_{o}∥{\overset{ˆ}{k}}_{\text{i}}$ and $B_{o}∥-{\overset{ˆ}{k}}_{\text{i}}$ orientation states are distinguishable in $Q_{b}$ only when $B_{o}$ is high, regardless of *T*. Also, $B_{o}∥{\overset{ˆ}{e}}_{\text{i}}$ and $B_{o}∥-{\overset{ˆ}{e}}_{\text{i}}$ orientation states, and $B_{o}∥{\overset{ˆ}{h}}_{\text{i}}$ and $B_{o}∥-{\overset{ˆ}{h}}_{\text{i}}$ orientation states are distinguishable in all scattering efficiencies, regardless of *T* and $B_{o}$.

Finally, results for dielectric-core and PEC-core cases show that the discrepancy among $Q_{s}(B_{o}∥{\overset{ˆ}{k}}_{\text{i}})$, $Q_{s}(B_{o}∥{\overset{ˆ}{e}}_{\text{i}})$, and $Q_{s}(B_{o}∥{\overset{ˆ}{h}}_{\text{i}})$ cases is strongly related to *T* and $B_{o}$, regardless of the frequency. This holds true for $Q_{a}$. For $Q_{b}$, on the other hand, this holds only when f>1.5 THz, regardless of *T* and $B_{o}$.

## CRediT authorship contribution statement

**Hamad M. Alkhoori:** Validation, Software, Project administration, Methodology. **Noura N. Alderei:** Writing – review & editing, Writing – original draft, Software, Investigation, Data curation. **Aryam S. Alshamsi:** Writing – review & editing, Writing – original draft, Visualization, Software, Data curation.

## Declaration of Competing Interest

The authors declare that they have no known competing financial interests or personal relationships that could have appeared to influence the work reported in this paper.
